# Object-based grouping benefits without integrated feature representations in visual working memory

**DOI:** 10.3758/s13414-020-02153-5

**Published:** 2020-10-18

**Authors:** Siyi Chen, Anna Kocsis, Heinrich R. Liesefeld, Hermann J. Müller, Markus Conci

**Affiliations:** Allgemeine und Experimentelle Psychologie, Department Psychologie, Ludwig-Maximilians-Universität München, Leopoldstr 13, D-80802 München, Germany

**Keywords:** Visual working memory, Kanizsa figure, Object-based representation, Feature-specific representation

## Abstract

Visual working memory (VWM) is typically considered to represent complete objects—that is, separate parts of an object are maintained as bound objects. Yet it remains unclear whether and how the features of disparate parts are integrated into a whole-object memory representation. Using a change detection paradigm, the present study investigated whether VWM performance varies as a function of grouping strength for features that either determine the grouped object (orientation) or that are not directly grouping relevant (color). Our results showed a large grouping benefit for grouping-relevant orientation features and, additionally, a much smaller, albeit reliable, benefit for grouping-irrelevant color features when both were potentially task relevant. By contrast, when color was the only task-relevant feature, no grouping benefit from the orientation feature was revealed both under lower or relatively high demands for precision. Together, these results indicate that different features of an object are stored independently in VWM; and an emerging, higher-order grouping structure does not automatically lead to an integrated representation of all available features of an object. Instead, an object benefit depends on the specific task demands, which may generate a linked, task-dependent representation of independent features.

Organizing the retinal image into coherent and meaningful objects is one of the fundamental operations of human vision. Gestalt principles, such as grouping by proximity, similarity, and good continuation, support the perceptual organization of the ambient array and make grouped objects appear to “belong together” and be processed as a whole. For example, in the Kanizsa figure (Kanizsa 1955; Fig. 1a, grouped), the presentation of Pac-Man-shaped inducer elements gives rise to the perception of an integrated star-shaped object, which is perceived as lying in front of the adjacent circular elements. Thus, in this example, spatial grouping processes effectively combine disparate parts to form a complete illusory figure.

**Fig. 1:**
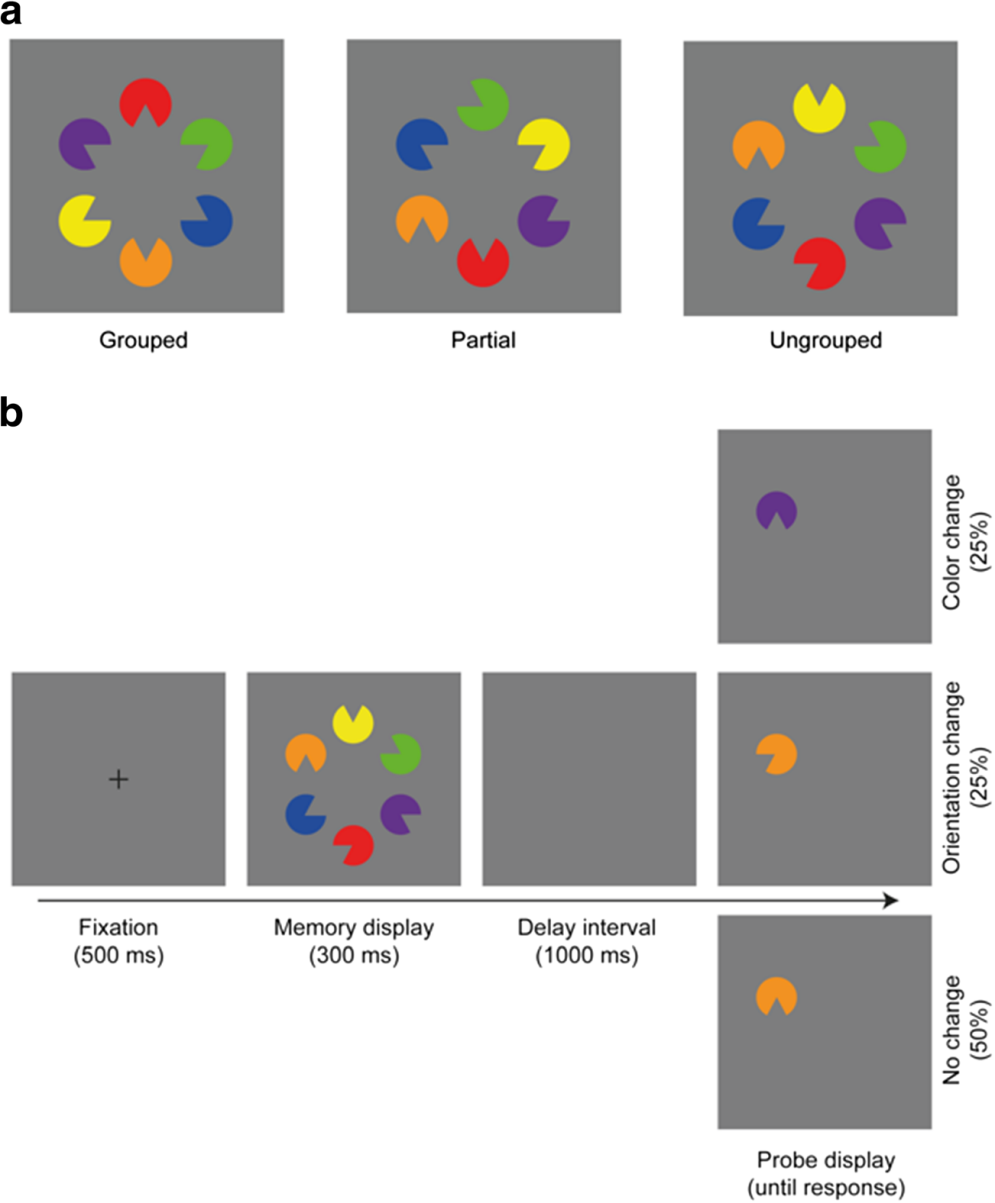
**a** Examples of the memory displays in the grouped (left), partially grouped (middle), and ungrouped (right) conditions. Note that each display presented exactly six different colors and orientations, such that the overall physical stimulation was identical in all three conditions. **b** Example trial sequence, depicting an ungrouped memory display, and corresponding illustrations of a color change (top right), an orientation change (middle right) and a no-change (bottom right) probe display. (Color figure online)

This operation of binding smaller units into integrated whole objects supports the efficient structuring of incoming sensory information, thereby reducing capacity limitations in visual working memory (VWM; Delvenne & Bruyer, 2006; Luck & Vogel, 1997; Morey, 2019; Morey, Cong, Zheng, Price, & Morey, 2015; Nie, Müller, & Conci, 2017; Peterson & Berryhill, 2013; Quinlan & Cohen, 2012; Vogel, Woodman, & Luck, 2001; Zhang & Luck, 2008). For instance, Brady and colleagues showed that to-be-remembered colors may be grouped, so that to-be-memorized items can be stored in compressed form, which releases capacity and permits more items to be memorized (Brady, Konkle, & Alvarez, 2009; Woodman, Vecera, & Luck, 2003). Similarly, Gao and colleagues (Gao, Gao, Tang, Shui, & Shen, 2016) showed that memorizing the orientation of a gap in disks was better when separate disks were grouped to form an illusory rectangle. This indicates that grouping operations increase the amount of information that can be stored in VWM. Moreover, there is evidence that VWM not only uses object grouping to compress the stored information to save capacity, but perceptual completion may conversely also improve the resolution of a memorized object—for example, when an occluded part of an object is completed behind an occluder to increase the fidelity of the object maintained in memory (Chen, Müller, & Conci, 2016; Chen, Töllner, Müller, & Conci, 2018b).

These findings support the idea that spatial grouping benefits VWM performance by integrating separate units into a complete-object representation, either leading to item compression with enhanced storage capacity, or more detailed, higher-resolution object representations. Besides grouping of spatially segregated items, individual nonspatial features (e.g., color and orientation) may also be bound into an integrated object. For instance, Luck and Vogel (1997) showed that VWM capacity was essentially independent of the to-be-memorized features that constituted a given object; rather, VWM performance was determined by the number of separate objects (see also Delvenne & Bruyer, 2004; Vogel et al., 2001; but see Wheeler & Treisman, 2002). This has been taken to suggest that features may be represented as bound objects in VWM (e.g., Luck & Vogel, 1997; Luria & Vogel, 2011; but see Gao, Gao, Li, Sun, & Shen, 2011; Ma, Husain, & Bays, 2014). Evidence for the object-based maintenance of visual information has also been reported in studies that compared feature-based processing with object-based processing. For instance, memorizing five colors or five orientations that appear in the same five objects has been reported to be easier than remembering the same 10 features in separate objects (Fougnie, Cormiea, & Alvarez, 2013; Olson & Jiang, 2002; Xu, 2002). Moreover, VWM performance has also shown to be enhanced when memory displays present three colors and three shapes that are combined to form three bound objects, compared with displays where the same three colors were presented in the background that surrounded each of the three shapes and are thus not bound to the object’s shape (Ecker, Maybery, & Zimmer, 2013). Together, these findings strengthen the view that VWM stores visual information in terms of integrated object representations.

Although VWM for a specific set of features can be improved by providing an integrated object representation, it is not clear whether all features of an object are actually bound into a unified representation. Recent evidence, in fact, suggests that different features are stored in a feature-specific memory format despite revealing complete-object benefits in VWM performance (Bays, Wu, & Husain, 2011; Fougnie & Alvarez, 2011; Fougnie et al., 2013; Magnussen & Greenlee, 1999; see also Pasternak & Greenlee, 2005, for a review). For instance, memory for one object feature was found to be relatively independent from other features from the same object (Fougnie et al., 2013), with each feature dimension having its own capacity limit and little cross-dimensional interference (Wang, Cao, Theeuwes, Olivers, & Wang, 2017). Given this, a memory benefit for integrated objects would not necessarily imply that all features of an object are actually stored in terms of an integrated, object-based representation. Instead, it is possible that a feature-specific representation at a basic level is bound to a complete object at a hierarchically higher representational level (Brady, Konkle, & Alvarez, 2011; Nie et al., 2017), thus bringing about the above-described object benefits in VWM based on an underlying hierarchical feature/object representation.

Taken together, the available evidence regarding the representation of visual information in working memory renders a rather heterogeneous picture: While some studies suggest that integrated objects can improve VWM performance, others point to a feature-based representational format. To investigate more directly how separate features of an object are represented in VWM, the current study employed a perceptual grouping manipulation, which promotes the integration of separate units into a whole object. For example, the percept of an illusory Kanizsa figure (see Fig. 1a, grouped) is achieved through grouping the Pac-Man inducers based on collinearity and closure (Chen, Glasauer, Müller, & Conci, 2018a; Conci, Müller, & Elliott, 2007). By contrast, no coherent object is rendered when the Pac-Man inducers are randomly oriented (see Fig. 1a, ungrouped). Thus, given that variations in the Pac-Man figure’s orientation determine whether or not a complete whole is formed, orientation can be considered a “grouping-relevant” feature for the stimulus configuration at hand. By contrast, other features of the Pac-Man figures, such as their color, exert no influence on the completed object representation and can therefore be considered “grouping irrelevant.” Previous studies mostly tested features that are directly relevant for grouping, with the results consistently showing that memory performance improves with an increase in grouping strength (e.g., Gao et al., 2016; see also Li, Qian, & Liang, 2018, for a recent meta-analysis of the effects of grouping on VWM accuracy measures). What remains unclear, however, is whether grouping can also enhance VWM for features that do not directly contribute to the grouped object. This was the question put to test in the present study—that is, how does perceptual grouping of parts into a complete illusory figure influence the retention of grouping-relevant and grouping-irrelevant features in VWM? For instance, grouping may result in a bound object, with separable parts being integrated into a coherent whole. When assuming an object-based memory representation, such a representation of an integrated whole should, in turn, enhance memory performance for both grouping-relevant and grouping-irrelevant features to a comparable extent. However, on the basis of a feature-based representation in VWM, one would assume that a completed object representation depends on the specific feature that affords grouping, then memory performance for grouping-relevant and grouping-irrelevant features should benefit to a variable degree from the integrated object structure. The latter would thus indicate that different features are—to some extent—maintained separately from each other in VWM.

To decide between these alternatives, we employed a change detection paradigm (Luck & Vogel, 1997). At the beginning of a trial, participants were presented with a memory display consisting of six Pac-Man figures, each with a unique color and orientation, that could be grouped to form a complete illusory star, or render a partially grouped triangle or, respectively, an ungrouped configuration—thus gradually manipulating the strength of the complete-object representation (see Fig. 1a; Chen, Nie, Müller, & Conci, 2019). Following a brief delay after memory display offset, a single Pac-Man probe item appeared at one of the locations that had been occupied by an item in the memory display. The task was to decide whether the probe item was the same as or different from the Pac-Man presented previously at the same location in the memory display (see Fig. 1b). Critically, the change could occur for a grouping-relevant feature (orientation), or for a grouping-irrelevant feature (color). Thus, by systematically varying closure in the Kanizsa-type configuration of the memory display (from a complete grouping through a partial grouping to an ungrouped configuration), we examined whether change detection performance would vary as a function of grouping strength for features that either did or did not determine the grouped object (orientation and color, respectively).

## Experiment 1a

The aim of Experiment 1a was to investigate how perceptual grouping influences the representation of various features of an object in VWM. We used a change-detection task to assess performance accuracy in memorizing the color and orientation of Pac-Man items that were presented either as a fully grouped, a partially grouped, or an ungrouped configuration. Note that the various Pac-Man arrangements produced configurations differing in grouping strength, however, without impacting the low-level properties of the image. On a given trial, both the color and the orientation of the Pac-Man inducers were potentially task relevant (with equal probability). If grouping improves VWM for both types of features to a similar degree, the benefit would likely be attributable to a single, unified representation of an integrated object in memory. However, if separable (grouping-driving and nondriving) features are differentially affected by grouping, they are likely maintained in separate, independent stores.

### Methods

#### Participants

Twenty volunteers (four males; mean age = 23.3 years) participated in Experiment 1a, either for payment of €8.00 per hour or for course credits. All participants had normal or corrected-to-normal vision, and all but one were right-handed. All observers provided written informed consent, and the experimental procedure was approved by the ethics committee of the Department of Psychology, Ludwig-Maximilians-University, Munich. The sample size was comparable to or larger than previous, similar studies (Fougnie et al., 2013; Gao et al., 2016). A power analysis conducted with G*Power (Erdfelder, Faul, & Buchner, 1996) revealed that to detect a relatively large effect, *f*(U) = 0.5, of object configuration with a power of 95% and an alpha of .05, a sample of only 12 participants would be required. We further increased our sample to *N* = 20 observers to ensure sufficient statistical power in our analyses.

#### Apparatus and stimuli

Stimuli were presented on a 24-inch TFT monitor. The experiment was implemented in MATLAB using Psychophysics Toolbox functions (Brainard, 1997). Participants were seated at a distance of approximately 57 cm from the screen.

The memory display consisted of six items, presented on an imaginary circle around the center of the screen (radius: 4° of visual angle), with all items arranged equidistantly to one another. Each item was a filled circle with a radius of 2.4° of visual angle and a 60° opening, thus forming a “Pac-Man”-like figure. Each Pac-Man was presented in a different color (blue, green, orange, red, purple, or yellow) and with a different orientation of its “mouth” (i.e., for a given Pac-Man, the cutout section could be rotated at an angle of 0°, 60°, 120°, 180°, 240°, or, respectively, 300°). The distribution of the six colors among the six items was randomized on every trial. The distribution of the “mouth” orientations was determined by the three experimental conditions that were presented with equal probability throughout the experiment. In the “ungrouped” condition, the six possible mouth orientations were randomly assigned to the six display locations. In the “partial” grouping condition, the openings of three items were oriented towards the center of the display, thus forming either an upward-pointing or downward-pointing (illusory) triangle. The mouth orientations of the other remaining three items were selected randomly from the remaining three orientations (without replacement of an already assigned orientation). Finally, in the “grouped” condition, the openings of all six items were oriented towards the center of the screen such that they formed an illusory star (see Fig. 1a for an illustration of the different object configurations). In this way, a given memory display would always consist of six distinct colors and six distinct mouth orientations, irrespective of the grouping condition. Thus, for all three types of configuration, each display presented an equal number of (six) colors and orientations, such that the basic physical stimulation was identical across conditions. Of note, the ungrouped configuration served as a baseline: the Pac-Man elements were randomly oriented (as well as randomly colored), making them unlikely to render any kind of grouped object. Moreover, for the six randomly selected colors and six randomly selected orientations, the random variation of the color and orientation features was comparable between the two to-be-memorized features. These random baseline configurations thus allowed us to assess whether change detection performance would be enhanced by any type of grouped structure.

#### Design and procedure

A trial started with the presentation of a central black fixation cross (0.6° × 0.6°) on a gray background (RGB: 125, 125, 125) for 500 ms. Next, the “memory display” appeared, presenting an ungrouped, partially grouped, or grouped configuration. The configuration was shown for 300 ms, after which only the gray background remained for a “delay period” of 1,000 ms. Next, a “probe display” appeared that consisted of a single Pac-Man item positioned randomly at one of the six possible item locations (that had been occupied in the memory array).^1^ In half of the trials, the probe was identical (in terms of both color and gap orientation) to the item presented at that particular location in the previous memory display (no-change condition). In the other half of trials, the probe item was changed in *either* color *or* orientation (with equal probability) relative to the probed item in the memory array. The change was realized by presenting the probed item in either the color or the orientation of one of the other five items (randomly selected) in the memory display, thus encouraging observers to memorize individual items as conjunctions of color and orientation (rather than just independent sets of orientations and colors). The probe display remained visible until the participant issued a response—namely, pressing the left or the right mouse key to indicate whether the probe item was the same as or different, respectively, from the Pac-Man at the same location in the preceding memory display. Participants were instructed to respond as accurately as possible. In case of an erroneous response, feedback was provided in the form of a red minus sign presented for 1,000 ms at the center of the screen. The next trial then started after an intertrial interval of 1,000 ms. Figure 1b illustrates an example trial sequence.

In total, every participant completed one practice block and 25 experimental blocks of 24 trials each. Trials were presented in randomized order such that all conditions—that is, the possible configurations (grouped, partially grouped and ungrouped) and change types (no change, color, or orientation change)—were presented randomly intermixed across trials. Thus, observers were required to memorize both the color and orientation features in the memory displays. The practice block was not included in the analyses. The actual experiment consisted of 600 trials in total. After each block, participants had the opportunity to take a short break; they moved on to the next block by pressing the space bar on the keyboard.

### Results

**Fig. 2:**
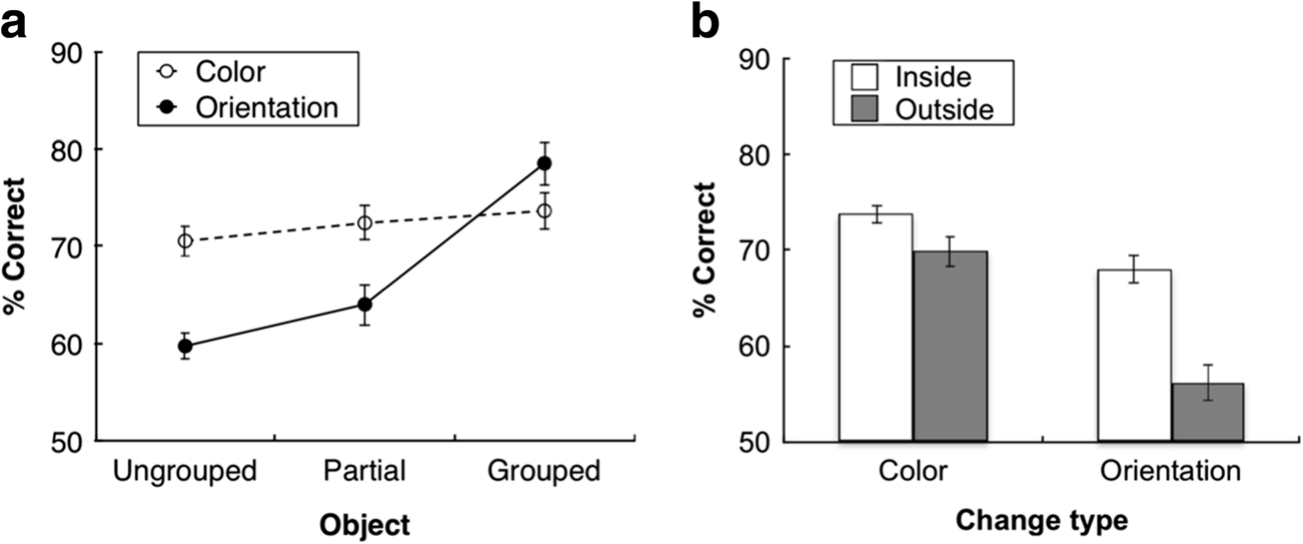
**a** Mean percentage of correct responses as a function of object configuration (grouped, partially grouped, and ungrouped) for the color and orientation changes in Experiment 1a. **b** Mean percentage of correct responses as a function of change type (color and orientation) in the partially grouped triangle condition of Experiment 1a. Accuracies in (**b**) are plotted separately for trials on which the probe was one of the three Pac-Man figures that gave rise to the illusory triangle (inside), or, respectively, on which the probe was one of the three nongrouped Pac-Man figures (outside). Error bars denote the within-subjects standard errors of the means

Figure 2a presents the percentage of correct responses as a function of object configuration, separately for color and orientation changes. To determine whether there were differences in accuracy across the different experimental conditions, we performed a repeated-measures analysis of variance (ANOVA) with the factors object configuration (grouped, partially grouped, ungrouped) and change type (color, orientation). We additionally report the estimated Bayes factors (BF_10_), obtained from comparable Bayesian statistics using JASP (Love et al., 2015). The Bayes factor provides the ratio with which the alternative hypothesis is favored over the null hypothesis (values below 1/3 may be taken to support the null hypothesis, whereas values greater than 3 would provide evidence in favor of the alternative hypothesis; see Jeffreys, 1961; Kass & Raftery, 1995). As we had a priori hypotheses about the direction of effects (we predicted that grouping leads to increased memory performance), one-tailed paired-samples *t* tests (along with one-tailed Bayesian paired-samples *t* tests) were used to compare different object configurations.

This analysis yielded significant main effects of object configuration, *F*(2, 38) = 38.85, *p* < .0001, η_p_^2^ = .67, 90% confidence interval, or CI [.50, .75], BF_10_ > 100, and of change type, *F*(1, 19) = 17.78, *p* < .0001, η_p_^2^ = .48, 90% CI [.19, .64], BF_10_ = 14.89. There was a graded effect of object configuration, with the highest accuracy for grouped configurations (76%), followed by partially grouped (68%) and ungrouped (65%) configurations. In addition, accuracy was higher for color changes than for orientation changes (72% and 67%, respectively). Finally, the Object Configuration × Change Type interaction was significant, *F*(2, 38) = 29.12, *p* < .0001, η_p_^2^ = .61, 90% CI [.41, .70], BF_10_ > 100, indicating that the enhancement of performance with increasing grouping strength was much larger for orientation changes (grouped vs. ungrouped: 19%, *p* < .001, *d*_z_ = 1.88, BF_10_ > 100; grouped vs. partially grouped: 15%, *p* < .001, *d*_z_ = 1.50, BF_10_ > 100; partially grouped vs. ungrouped: 4%, *p* = .019, *d*_z_ = 0.5, BF_10_ = 3.41) than for color changes (grouped vs. ungrouped: 3%, *p* = .009, *d*_z_ = 0.59, BF_10_ = 6.53; grouped vs. partially grouped: 1%, *p =* .18, *d*_z_ = 0.21, BF_10_ = 0.56; partially grouped vs. ungrouped: 2%, *p* = .02, *d*_z_ = 0.49, BF_10_ = 3.04). It should be noted, however, that both types of change benefited significantly (albeit to a differential degree) from the increase in grouping strength.^2^ Overall, the mean performance in this experiment was around 70%, while decreasing in some conditions to ~60% (e.g., in the orientation change condition with ungrouped configurations). Importantly, though, the mean accuracies were significantly above chance level in all conditions, *t*s(19) > 6.54, *p*s < .001, *d*s > 1.46, BF_10_s >100 (this was also the case in all subsequent experiments).

Given these effects of grouping on the mean change-detection accuracies, we went on to examine whether the presence of a grouped object would also reduce the amount of guessing reflected in the false-alarm (FA) rates (i.e., “change” responses when there was actually no change on a given trial). A repeated-measures ANOVA of the FA rates with the factors object configuration and change type revealed a significant main effect of configuration, *F*(2, 38) = 22.00, *p* < .001, η_p_^2^ = .54, BF_10_ > 100. The FA rate was significantly lower for the grouped configuration (28%) versus both the ungrouped (39%) and partially grouped (36%) configurations, which showed a comparable amount of guessing (grouped vs. partially grouped, *p* < .001, *d*_*z*_ = −1.40, BF_10_ > 100; grouped vs. ungrouped, *p* < .001, *d*_*z*_ = −1.23, BF_10_ > 100; ungrouped vs. partially grouped, *p* = .19, *d*_*z*_ = −0.30, BF_10_ = 0.50). Thus, the complete star grouping led not only to improved performance overall but also to a substantial reduction of guessing. The main effect of change type and the two-way interaction were not significant, change type: *F*(1, 19) < 1, *p* > .99, η_p_^2^ = 0, BF_10_ = 0.19; Object Configuration × Change Type interaction, *F*(2, 38) = 2.61, *p* = .086, η_p_^2^ = .12, BF_10_ = 0.54, indicative of a relatively constant rate of guessing across the two types of feature change (grouped, partially grouped, ungrouped: 29%,: 35%, and 39%, respectively, for color changes; 27%, 38%, and 38%, respectively, for orientation changes).

In a further analysis, we examined whether the overall accuracies and the associated grouping effects changed with increasing time on task. To this end, the data from each five consecutive blocks were averaged into one epoch, resulting in five epochs overall. A repeated-measures ANOVA of change-detection accuracy with the factors epoch, object configuration, and change type failed to reveal any significant effects involving epoch, main effect: *F*(4, 76) = 0.42, *p* = .79, η_p_^2^ = .02, BF_10_ < 0.01; Epoch × Object Configuration and Epoch × Object Configuration × Change Type interactions: both *p*s > .61, η_p_^2^s < .04, BF_10_s < 0.01. This indicates that performance was relatively stable overall, with the benefit of grouping being evident right from the beginning of the experiment, and without revealing major variations throughout the entire duration of the experiment (there was also no variation in performance across epochs in the subsequent experiments). All other significant effects mirrored the pattern of results described above.

Finally, a subsequent analysis then examined whether change detection performance was influenced by the probe location in partially grouped displays (with triangle groupings). Figure 2b presents the percentage of correct responses for color and orientation changes, separately for trials on which the probe was presented at one of the three Pac-Man locations that formed the illusory triangle (inside) and trials on which the probe appeared at one of the three other, “nongrouped” Pac-Man figures (outside). A two-way repeated-measures ANOVA of the accuracies in the partially grouped triangle configuration, with the factors change type (color, orientation) and probe location (inside, outside), revealed both main effects to be significant: change type, *F*(1, 19) = 29.34, *p* < .0001, η_p_^2^ = .61, 90% CI [.33, .73], BF_10_ > 100; probe location, *F*(1, 19) = 22.65, *p* < .0001, η_p_^2^ = .54, 90% CI [.25, .69], BF_10_ = 36.02. Accuracies were higher for color changes (72%) than for orientation changes (62%), mirroring the analysis described above. In addition, the accuracies were increased when the probe was presented inside the partially grouped triangle (71%) as compared with an outside location (63%). The Change Type × Probe Location interaction was marginally significant, *F*(1, 19) = 3.95, *p* = .06, η_p_^2^ = .17, 90% CI [.00, .39], BF_10_ = 1.89, reflecting a tendency for the inside-triangle benefit to be larger for orientation changes (12%, *p* < .0001, *d*_z_ = 0.88, BF_10_ = 81.35) than for color changes (4%, *p* = .04, *d*_z_ = 0.41, BF_10_ = 1.78), though accuracy was significantly better for both orientation and color changes when they were part of the illusory triangle.

### Discussion

Experiment 1a demonstrates that change detection performance is modulated both by grouping strength and the type of feature change that was to be detected. Performance was more accurate with greater grouping strength in the memory display, and changes in color were overall easier to detect than changes in orientation. Importantly, however, both color and orientation features benefited significantly from grouping—albeit to variable degrees: The grouping benefit was larger for changes in orientation than for changes in color (for the latter, there was only a small, though reliable improvement with increased grouping strength). A comparable variation in performance with grouping strength was also revealed in an additional analysis of the partially grouped displays, which showed that accuracy was higher when to-be-detected changes occurred in grouped (inside-triangle) compared with nongrouped (outside-triangle) items of the very same configuration. In this analysis, too, color changes exhibited smaller grouping benefits than orientation changes. Together, this pattern of results indicates that although grouping facilitates both grouping-relevant and grouping-irrelevant features, features that come to be integrated to form an emergent object yield a much larger benefit than features that are not an intrinsic part of the grouped spatial structure.

## Experiment 1b

Experiment 1a showed that perceptual grouping can improve VWM for both color and orientation information. However, the fully grouped configuration presumably not only integrates the presented parts into a coherent, “whole” object, but it also presents a symmetric and regular organization of the individual Pac-Man elements. Thus, the observed enhancement of VWM performance could potentially have resulted from two orthogonal processes that could potentially both impact performance: the integration of disparate items into a whole-object grouping and the organization of the display in terms of a regular and symmetric item layout.

**Fig. 3:**
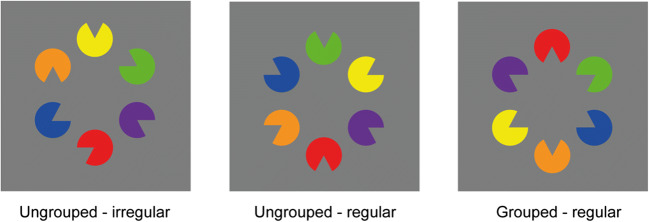
Examples of the memory displays in the ungrouped/irregular (left), ungrouped/regular (middle), and grouped/regular (right) configurations. The ungrouped/irregular and grouped/regular configurations were identical to the ungrouped and grouped stimulus conditions presented in Experiment 1a (see Fig. 1). (Color figure online)

Given this, Experiment 1b was performed to determine the contribution of the effect of regularity upon object grouping. To this end, the grouped “star” shape memory display (grouped/regular; see Fig. 3, right) was now compared with two variants of ungrouped memory displays: one which provided an irregular configuration (grouped/irregular; see Fig. 3, left) comparable with the ungrouped configurations presented in Experiment 1a, and one which depicted an ungrouped but nevertheless regular—that is, symmetrical configuration (ungrouped/regular; see Fig. 3, middle). If working memory for orientation and color primarily benefits from the regularity of the presented configuration, then performance in the new ungrouped/regular condition should be comparable to the grouped (star) condition, because both depict equally regular and symmetric item arrangements (relative to the ungrouped/irregular baseline). However, if we nevertheless found a reliable advantage for the star configuration, relative to the ungrouped/regular configuration, this would argue in favor of a VWM-related grouping benefit (over and above the availability of a regular structure, which might also impact performance).

### Methods

Experiment 1b was in essence identical to Experiment 1a, except that the partially grouped configuration was replaced by a new “ungrouped/regular” configuration, which was compared with the “grouped/regular” and “ungrouped/irregular” configurations (the latter being identical to the grouped and ungrouped configurations of Experiment 1a; see example configurations in Fig. 3). In this new, ungrouped/regular configuration, all six Pac-Man figures were presented with randomly assigned colors but with fixed orientations, with the mouth of each Pac-Man facing away from the center of the display configuration, thereby generating a regular and symmetric organization that, however, lacked a grouped illusory object (see Fig. 3, middle). All experimental conditions were again presented in random order across trials. A new group of 14 observers (six males; mean age = 26.8 years) participated in Experiment 1b, thus conforming to the abovementioned power estimates. All participants were right-handed and they all had normal or corrected-to-normal vision. All other details were the same as described for Experiment 1a.

### Results

**Fig. 4:**
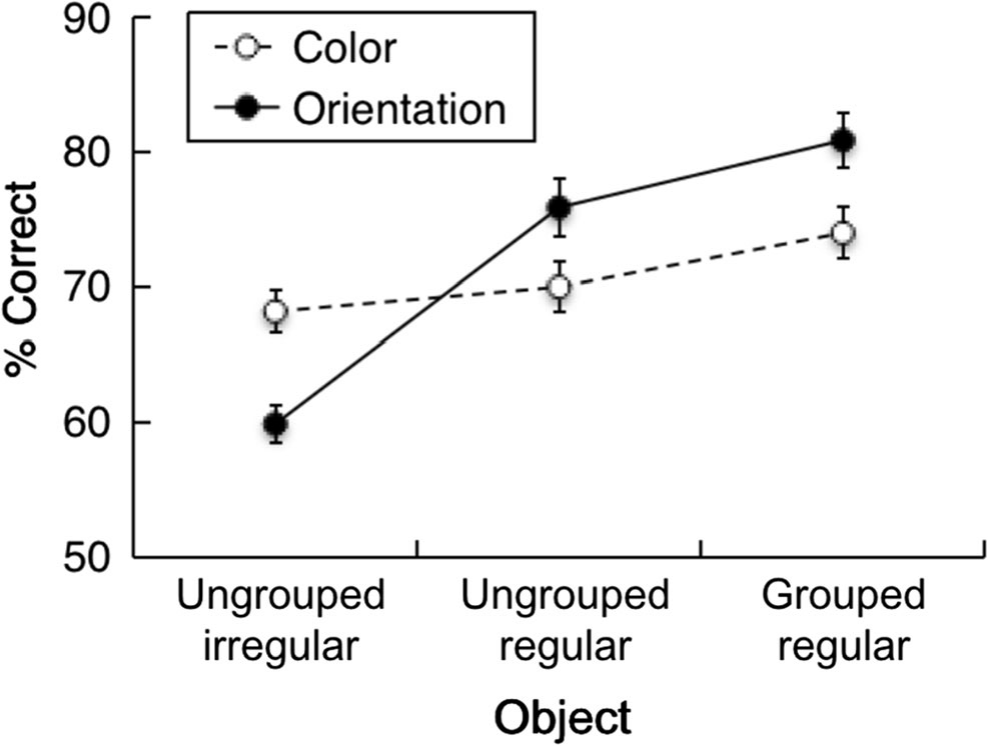
Mean percentage of correct responses as a function of object configuration (ungrouped/irregular, ungrouped/regular, and grouped/regular), separately for color and orientation changes in Experiment 1b. Error bars denote the within-subject standard errors of the means

Figure 4 presents the percentage of correct responses as a function of object configuration, separately for color and orientation changes. A repeated-measures ANOVA with the factors object configuration (grouped/regular, ungrouped/regular, ungrouped/irregular) and change type (color, orientation) yielded a significant main effect of object configuration, *F*(2, 26) = 62.06, *p* < .001, η_p_^2^ = .83, BF_10_ > 100, with the highest accuracy for the grouped/regular configuration (78%), followed by the ungrouped/regular (73%) and ungrouped/irregular (64%) configurations. The main effect of change type was not significant, *F*(1, 13) = 1.72, *p* = .21, η_p_^2^ = .12, BF_10_ = 0.29. However, the Object Configuration × Change Type interaction was significant, *F*(2, 26) = 79.05, *p* < .001, η_p_^2^ = .86, BF_10_ > 100. Decomposing this interaction by post hoc tests revealed that for *color changes*, there was a marginally significant difference between ungrouped/irregular and ungrouped/regular configurations, mean difference: 1.8%, *t* (13) = −1.64, *p* = .06, *d*_z_ = 0.44, BF_10_ = 1.47, whereas the grouped/regular configuration yielded a higher accuracy compared with both the ungrouped/irregular and the ungrouped/regular configurations, grouped/regular vs. ungrouped/irregular: 6%, *t*(13) = 4.81, *p* < .001, *d*_z_ = 1.29, BF_10_ > 100; grouped/regular vs. ungrouped/regular: 4%, *t*(13) = 6.66, *p* < .001, *d*_z_ = 1.78, BF_10_ > 100. For *orientation changes*, performance was the highest for the grouped/regular configuration, followed by the ungrouped/regular configuration, and being the lowest for the ungrouped/irregular configuration, grouped/regular vs. ungrouped/irregular: 21%, *t*(13) = 9.89, *p* < .001, *d*_z_ = 2.64, BF_10_ >100; grouped/regular vs. ungrouped/regular: 5%, *t*(13) = 5.03, *p* < .001, *d*_z_ = 1.34, BF_10_ > 100; ungrouped/regular vs. ungrouped/irregular: 16%, *t*(13) = 8.91, *p* < .001, *d*_z_ = 2.38, BF_10_ > 100. Thus, while there was a rather large benefit of regular organization for orientation changes, there was only a small, statistically marginal benefit of regularity for color changes. Importantly, however, an additional benefit of object grouping was found for color as well as for orientation changes with grouped/regular configurations (relative to both types of ungrouped configurations, whether irregular or regular)—thus representing a genuine effect of (object) grouping.

A subsequent examination of the FA rates, by means of a repeated-measures ANOVA with the factors object configuration and change type, revealed a significant main effect of configuration, *F*(2, 26) = 39.73, *p* < .001, η_p_^2^ = .75, BF_10_ > 100: FA rates were highest for ungrouped/irregular (46%), intermediate for ungrouped/regular (36%), and lowest for grouped/regular configurations (28%; all *p*s <.001, *d*_*z*_s > 1.30, BF_10_s > 100). As in Experiment 1a, the effect of change type and the two-way interaction were nonsignificant, change type: *F*(1, 13) = 0.19, *p* = .67, η_p_^2^ = .02, BF_10_ = 0.28; Object Configuration × Change Type interaction, *F*(2, 26) = 2.68, *p* = .088, η_p_^2^ = .17, BF_10_ = 1.17. This pattern indicates that the effects of regularity and grouping both come to reduce the rate of guessing, with comparable effects across change types (grouped/regular, ungrouped/regular, and ungrouped/irregular: 30%, 35%, and 46%, respectively, for color changes; 27%, 36%, and 47%, respectively, for orientation changes).

### Discussion

Experiment 1b was performed to dissociate the influence of perceptual grouping from configural regularity. The results demonstrated for equally regular configurations a reliable advantage for the grouped/regular star-shaped object relative to the ungrouped/regular, symmetric configuration, which was evident for both grouping-relevant (orientation) and grouping-irrelevant (color) features. This pattern argues in favor of some extra facilitation observed with grouped/regular configurations being the result of a genuine grouping-based representation of this configuration in VWM. Thus, this shows that object grouping ultimately resulted in some “linked” representation of both color and orientation features, thus in turn revealing a combined benefit of grouping that is specific to whole objects. However, the presentation of a regular structure (without concurrent shape grouping) also had a significant benefit for VWM performance: a marked benefit for detecting orientation changes and a lesser one for detecting color changes. This points to the involvement of some additional factor associated with configuration regularity, which also facilitates performance, over and above the effect of grouping. Thus, it appears that both object grouping and regularity can substantially benefit VWM performance, thus showing that various types of structured representations can enhance working memory. However, it should be noted that it is difficult to disentangle the relative impact of regularity and grouping, because the regular and symmetric arrangement of the grouped star object might also further enhance processing beyond a simple, purely additive influence. Finally, the results also replicated the overall findings of Experiment 1a, in showing better performance with grouped/regular versus ungrouped/irregular configurations for both color and orientation changes, with the grouping benefit being larger for orientation than for color changes.

Moreover, the presence of a regular display layout did also facilitate the detection of orientation changes, without having a comparable effect for color changes. This indicates that all features of an integrated object (i.e., in our case: orientation and color) may benefit from grouping—that is, from the formation of an object (at least to some extent); in contrast, benefits resulting from display regularity are largely confined to the feature that determines the regular structure (orientation). That is, grouping benefits in the orientation change-detection task might always incorporate some “hidden” effect of display regularity, which additionally enhances memory performance.

Finally, as noted above, in both Experiments 1a and 1b, the overall level of performance was relatively low (though significantly above chance level in all conditions). An obvious reason for this might be that the task was actually quite difficult, requiring observers to memorize six different colors and six different orientations—which is clearly above the typical estimate of three to four items that have been reported as a VWM capacity limit (Luck & Vogel, 1997). Despite this, our results for the ungrouped configurations (for which performance was the lowest) are still roughly comparable with previous working-memory studies that also used displays with six distributed items—for instance, an accuracy of ~63% for our ungrouped configurations compares with ~70% in Luck and Vogel (1997; see also Vogel et al., 2001), where the minor difference may be attributable to different types of probe employed (cf. Jiang et al., 2000). Thus, the overall low level of performance is consistent with previous work and appears to reflect the actual difficulty of the task.

## Experiment 2

Experiments 1a and 1b revealed an object-grouping benefit for both grouping-relevant (orientation) and grouping-irrelevant (color) features (and an additional benefit of configural regularity which arose mainly with the grouping-relevant orientation features). Importantly, in both experiments, the two types of changes were randomly intermixed within trial blocks and were therefore not predictable such that both the color and orientation features had to be memorized on a given trial. It might thus be possible that processing of the grouping-relevant features facilitated processing of the grouping-irrelevant features in particular because both features were potentially task-relevant and had to be attended/encoded.

Given this, Experiment 2 was designed to examine whether an overall VWM benefit of grouping would still be obtained when different features are uniquely task-relevant in separate parts of the experiment. To this end, in Experiment 2, color and orientation changes were implemented in separate trial blocks. If the benefit of grouping for color features evident in Experiment 1 was mainly due to specific task demands (i.e., the requirement to memorize all features of the presented configurations), then the grouping benefit for color changes should disappear under conditions in which the grouping-relevant feature does not need to be memorized.

### Methods

Experiment 2 was essentially the same as Experiment 1a, except that color and orientation changes were presented in two separate experimental halves. That is, only color changes or, respectively, only orientation changes were presented within a given half (along with 50% no-change trials), with condition order counterbalanced across participants. A new group of 20 volunteers (seven males; mean age = 24.4 years) was tested—comparable to the sample in Experiment 1a. All but three participants were right-handed and all had normal or corrected-to-normal vision. Each (color change and orientation change) half of the experiment started with 24 practice trials, followed by 12 experimental blocks of 24 trials, amounting to 576 experimental trials in total.

### Results

**Fig. 5:**
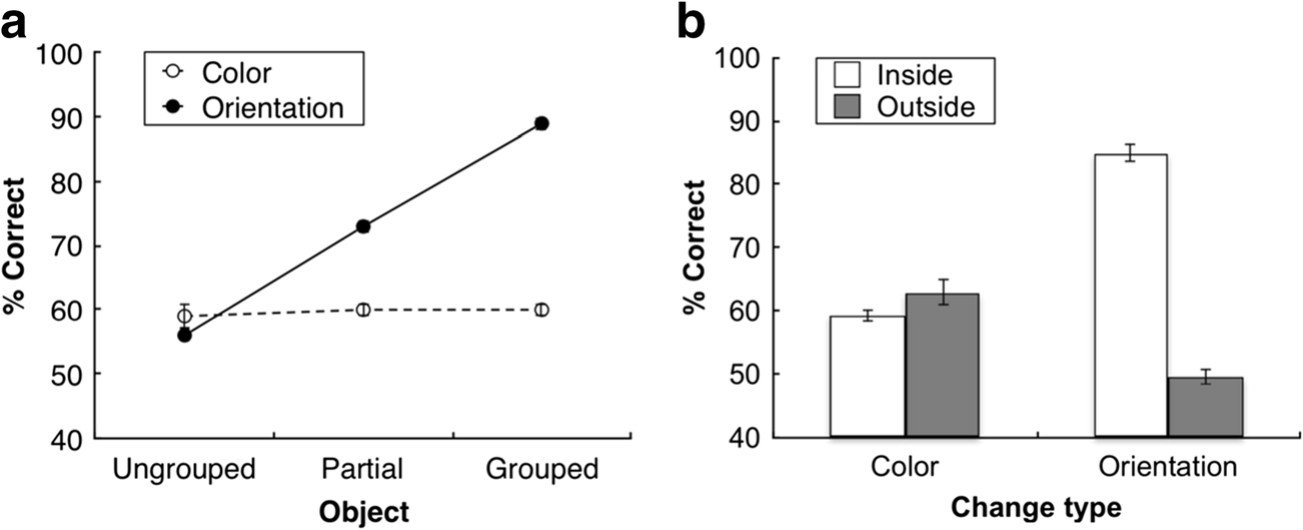
**a** Mean percentage of correct responses as a function of the object configuration (grouped, partially grouped, and ungrouped configurations), separately for color and orientation changes in Experiment 2. **b** Mean percentage of correct responses as a function of change type (color or orientation) separately for trials on which the probe was presented inside and, respectively, outside the partially grouped triangle in Experiment 2. Error bars denote the within-subject standard errors of the means

Figure 5a presents the percentage of correct responses as a function of object configuration, separately for the different change types. A two-way repeated-measures ANOVA on the accuracy data, with the factors object configuration (grouped, partially grouped, ungrouped) and change type (color, orientation), revealed both main effects to be significant: object configuration, *F*(2, 38) = 168.25, *p* < .0001, η_p_^2^ = .90, 90% CI [.84, .92], BF_10_ > 100; and change type, *F*(1, 19) = 103.13, *p* < .0001, η_p_^2^ = .84, 90% CI [.70, .89], BF_10_ > 100. There was again a graded effect of object configuration, with the highest accuracy for grouped configurations (75%), followed by partially grouped (67%) and ungrouped (58%) configurations. In addition, and in contrast to Experiment 1a, orientation changes were detected more accurately than color changes (73% and 60%, respectively). Finally, the interaction was also significant, *F*(2, 38) = 109.44, *p* < .0001, η_p_^2^ = .85, 90% CI [.76, .89], BF_10_ > 100. An increase in performance with increasing grouping strength was evident for the orientation changes, revealing a maximal increase (between grouped and ungrouped configurations) of 33% due to grouping (and its concurrent effect of regularity, see Experiment 1b; *p* < .0001, *d*_z_ = 4.23; grouped vs. partially grouped: 16%, *p* < .0001, *d*_z_ = 2.66; partially grouped vs. ungrouped: 17%, *p* < .0001, *d*_z_ = 2.52; all BF_10_ > 100). By contrast, there was no significant improvement for the color changes (grouped vs. ungrouped: 0.6%, *p* = .34, *d*_z_ = 0.09, *BF*_*10*_ = 0.33; grouped vs. partially grouped: −0.7%, *p* = .70, *d*_z_ = −0.12, BF_10_ = 0.16; partially grouped vs. ungrouped: 1.3%, *p* = .14, *d*_z_ = 0.25, BF_10_ = 0.70).

A subsequent ANOVA of the FA rates, with the factors object configuration and change type, revealed a significant main effect of configuration, *F*(2, 38) = 116.98, *p* < .001, η_p_^2^ = .86, BF_10_ > 100: the FA rate was highest for ungrouped (59%), intermediate rate for partially grouped (49%), and lowest for grouped configurations (34%; all *p*s <.001, *d*_*z*_s > 1.39, BF_10_s > 100). There was also a main effect of change type, *F*(1, 19) = 159.39, *p* < .001, η_p_^2^ = .89, BF_10_ > 100, with a higher rate of false color (59%) than false orientation responses (36%). The Object Configuration × Change Type interaction was also significant, *F*(2, 38) = 86.08, *p* < .001, η_p_^2^ = .82, BF_10_ > 100, while false color responses were comparable across all object configurations (grouped: 57%; partially grouped: 58%; ungrouped: 61%; ungrouped vs. partially grouped, *p =* .87, *d*_*z*_ = 0.25, BF_10_ = 0.52; ungrouped vs. grouped, *p =* .64, *d*_*z*_ = 0.35, BF_10_ = 1; grouped vs. partially grouped, *p =* .99, *d*_*z*_ = −0.09, BF_10_ *=* 0.28), false orientation responses were modulated by the strength of grouping (i.e., the stronger the grouping, the fewer orientation FAs; grouped: 11%; partially grouped: 39%; ungrouped: 57%; all *p*s <.001, *d*_*z*_s > 1.70, BF_10_s > 100). Thus, the analysis of the false alarms essentially replicates the pattern of results as observed for the accuracies.

In a subsequent analysis, we again examined whether the probe location in partially grouped (triangle) displays did influence change detection performance. Figure 5b presents the percentage of correct responses as a function of change type, separately for probes located inside the triangle grouping and probes located outside. A two-way repeated-measures ANOVA on the accuracies in the partially grouped triangle configuration, with the factors change type (color, orientation) and probe location (inside, outside) revealed all effects to be significant: change type, *F*(1, 19) = 10.30, *p* = .005, η_p_^2^ = .35, 90% CI [.08, .55], BF_10_ = 1.12; probe location, *F*(1, 19) = 63.40, *p* < .0001, η_p_^2^ = .77, 90% CI [.56, .84], BF_10_ > 100; Change Type × Probe Location interaction, *F*(1, 19) = 232.69, *p* < .0001, η_p_^2^ = .92, 90% CI [.85, .95], BF_10_ > 100. As already described above, accuracy was overall higher for orientation changes (67%) than for color changes (61%), and when an item that was part of the grouped triangle (72%) was probed as compared with an item from the other, ungrouped stimuli (56%). In addition, for color changes, the inside/outside difference was non-significant—if anything, there was a numerical disadvantage of −4%, *t*(19) = −1.38, *p* = .91, *d*_z_ = −0.31, BF_10_ = 0.11; for orientation changes, by contrast, there was a very robust, 35% advantage for grouped versus nongrouped locations, *t*(19) = 17.95, *p* < .0001, *d*_z_ = 4.02, BF_10_ > 100. Thus, blocking the type of change led to a large benefit of grouping, but this occurred only when observers needed to memorize grouping-relevant (orientation) features.

#### Cross-experiment comparisons

Comparing the results from Experiments 1a and 1b with Experiment 2, it appears that for the color change condition, performance dropped considerably with the blocked presentation in the latter experiment. This observation was confirmed by a mixed-design ANOVA on accuracy in the color change-detection task, with the within-subject factor object configuration (grouped, partially grouped, ungrouped) and the between-subject factor experiment (1a, 2—corresponding to mixed vs. blocked change types). The main effect of experiment was significant, *F*(1, 38) = 36.12, *p* < .001, η_p_^2^ = .49, BF_10_ = 1, reflecting overall higher performance in Experiment 1a than in Experiment 2 (mean difference: 13%). The effect of object configuration, *F*(2, 76) = 2.86, *p =* .06, η_p_^2^ = .07, BF_10_ < 0.01, and the two-way interaction, *F*(2, 76) = 1.05, *p =* .35, η_p_^2^ = .03, BF_10_ = 0.26, were nonsignificant. A comparable drop (of ~15%) in the detection of color changes was also observed for the ungrouped and grouped configurations from Experiment 1b to Experiment 2 (both *p*s < .001, *d*s > 0.7, BF_10_s > 100), whereas there were no differences when comparing Experiments 1a and 1b (both *p*s > .35, *d*s < 0.06, BF_10_s < 0.46, for ungrouped and grouped configurations).

For orientation changes, there were no significant differences when comparing performance for the ungrouped configurations across Experiments 1a, 1b, and 2 (Experiment 1a vs. Experiment 1b, *p =* .99, *d*_*z*_ < 0.01, BF_10_ *=* 0.33; Experiment 1a vs. Experiment 2, *p =* .64, *d*_*z*_ = 0.21, BF_10_ *=* 1; Experiment 1b vs. Experiment 2, *p =* .73, *d*_*z*_ = 0.19, BF_10_ = 1), thus indicating that baseline performance was overall comparable. In contrast, for the grouped configurations, performance was actually increased in Experiment 2 relative to Experiments 1a and 1b (both *p*s < .018, *d*s > 0.44, BF_10_s > 12.7). This is indicative of increased benefits from grouping when only the grouping-relevant (orientation) feature is task relevant.

### Discussion

The results of Experiment 2 again showed that grouping enhances detection accuracy, thus replicating the basic pattern of results of Experiments 1a and 1b. However, unlike Experiments 1a and 1b, in Experiment 2 there was a very marked benefit of grouping (and of configural regularity), but this was evident only for orientation changes, not for color changes. In fact, the grouping benefit was found to be even larger when the task required observers to maintain only the (grouping-relevant) orientations of the presented configurations (in Experiment 2), as compared with when they had to maintain both orientation and color to perform the task (in Experiments 1a and 1b). Also, for partially grouped configurations, the accuracy for orientation changes was again much higher when the probe was presented inside rather than outside the grouped triangle. By contrast, no such modulation of performance was observed for color changes. This suggests that spatial grouping only modulates performance when the grouping-relevant feature (orientation) is task-relevant; when orientation information is not directly task-relevant, grouping by orientation will not benefit change detection of other features, such as the object’s colors. Accordingly, the (small) increase in memory performance for the grouping-irrelevant (color) feature observed in Experiments 1a and 1b would be attributable to the fact both color and orientation features were simultaneously task-relevant in these experiments.

The observed lack of a grouping-dependent improvement in color change detection in Experiment 2 is overall consistent with a recent report by Li et al. (2018). They investigated whether real-contour and illusory-contour groupings can facilitate VWM for a grouping-irrelevant color feature in displays with three to five elements that were grouped to form triangles, squares, or pentagons. As in the current experiment, they found no reliable grouping benefit relative to an ungrouped baseline condition. In two further experiments, Li et al. (2018) varied the composition of the memory displays, now presenting displays in which some elements grouped to form a Kanizsa triangle, while others did not group. With this type of display, a significant grouping benefit emerged for grouping-irrelevant color changes. At a first glance, this manipulation is very similar to our “partially grouped” condition, which also consisted of both grouped and ungrouped elements (in the very same display)—however, for which we found no effect with blocked presentations. Of note, though, there was a crucial difference between the two experiments, which relates to the composition of the probe displays (to which a response had to be issued). In our experiments, only a single Pac-Man item was presented in the probe display, thus ruling out any possible impact of grouping on the processing of the probe display. In Li et al.’s (2018) experiments, by contrast, the very same item configuration from the memory display was again presented in the probe display (except for a single Pac-Man item that could have changed in color). While such “whole-probe” variants of the change-detection task usually lead to overall improved performance compared with single-item probes (see Jiang et al., 2000), they come with a disadvantage—namely, any effect of the display composition could potentially influence (i) the retention of items in working memory (i.e., following the presentation of the to-be-memorized items in the memory display) and/or (ii) the selection of a response (when all items are presented again, together with the changed item in the probe display). Thus, the improved performance for the grouped items in Li et al.’s (2018) study may not necessarily be due to a grouping-induced bias in working memory itself, but could have resulted from an attentional bias that arose when processing the final probe display to select a response. The latter would be consistent with several other studies that demonstrated an attentional bias towards Kanizsa-figure groupings in visual search tasks (e.g., Marini & Marzi, 2016; Rauschenberger & Yantis, 2001; Senkowski, Röttger, Grimm, Foxe, & Herrmann, 2005; Wiegand et al., 2015). Importantly, given that we presented a single item in isolation in the probe display, such an attentional bias operating at the response selection stage was effectively ruled out—and no grouping benefit was observable in the results of Experiment 2.

Another—at first glance—somewhat peculiar observation was that performance in the color change-detection task dropped considerably (by some 13 %) from Experiments 1a and 1b to Experiment 2, even though in the latter experiment, the working-memory load was reduced, requiring only one feature to be retained at any moment, as opposed to two task-relevant features in Experiments 1a and 1b. Potentially, this drop of performance has to do with a change in attentional focus, which is associated with the change in task demands. In Experiments 1a and 1b, the task was to memorize a conjunction of features (i.e., both the color and the orientation of the Pac-Man elements). That is, with this task setting, observers would need to memorize the “whole” display, which could be expected to engender a more “global” (object-based) processing mode. In Experiment 2, only one specific feature was task-relevant at any given moment and this in turn may result in a more “local” representation of the specific features that have to be retained. This change in task demands might thus have changed the scope of processing from a more global focus on the ensemble/object structure to a local, feature-specific representation—which could explain the overall reduced color-change detection performance in Experiment 2 relative to Experiments 1a and 1b (even though this reduction is in some way counterintuitive, given that the memory load was actually higher in Experiments 1a and 1b than in Experiment 2).

## Experiment 3

The experiments reported thus far indicate that grouping-relevant and grouping-irrelevant features both benefit (at least to some extent) from the integration of disparate items into a completed (illusory) object when both are relevant for the (change detection) task (Experiments 1a and 1b). In contrast, if only one of the two features is task-relevant, then only the grouping-relevant orientation feature engenders a performance benefit (Experiment 2). This indicates that separable features of an object are, to some extent, represented independently of each other, and memory for the grouping-irrelevant features will only benefit from grouping when the grouping-relevant features are task-relevant. It may be argued, however, that the specific task used in Experiments 1 and 2—a change-detection task that presented a discrete set of colors and required only a basic, two-alternative forced-choice response—impeded the storage of items as complete object representations, or that the effects of grouping are sometimes so subtle that they escape the rather coarse, binary measure (correct/incorrect) obtained in the change-detection task. Given this, Experiment 3 was performed to corroborate that grouping does not modulate performance when the grouping-relevant feature is not directly task-relevant, using a delayed estimation (rather than a change detection) task (e.g., Zhang & Luck, 2008). That is, participants were required to encode the (grouping-irrelevant) color of the stimuli presented in the memory display with high precision so as to be able to reproduce the exact color of the indicated item (in the probe display) on a color wheel, thus yielding a fine-grained measure of memory precision. This permitted us to test whether (spatial) grouping effects on grouping-irrelevant color features would come to the fore only if a sufficiently high degree of memory precision is required by the task.

### Methods

**Fig. 6:**
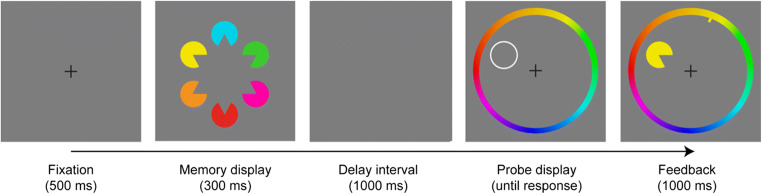
Example trial sequence in Experiment 3, depicting a grouped memory display, and a corresponding illustration of a color-wheel probe display, followed by a feedback display presenting the actual to-be-recalled Pac-Man together with a pointer highlighting the correct response on the color circle. (Color figure online)

In Experiment 3, a new group of 20 volunteers (10 males; mean age = 25.35 years) was tested. All but one of the participants were right-handed, and they all had normal or corrected-to-normal vision. Experiment 3 was essentially the same as the color-change condition in Experiment 2, except that we used 360 colors and the probe display consisted of an outline (placeholder) circle at the location of the item in the memory display whose color was to be reproduced, along with a “surrounding” color wheel centered at the monitor midpoint. Participants responded by clicking on their chosen color on the wheel using the computer mouse. Following the response, feedback was provided for 1,000 ms, displaying the actual Pac-Man that was to be recalled and a pointer at the location of the correct color on the wheel (see Fig. 6).

The color wheel had a radius of 8° and a thickness of 1° of visual angle, and consisted of 360 color values evenly distributed along a circle in the CIE L*a*b* color space, centered in one luminance plane (L = 63) at (a = 9, b = 27) with a radius of 40. The colors of the items in the memory display were randomly selected from the available set of colors. The color wheel was additionally presented at a random rotation on each trial to minimize contributions from spatial memory. Different types of (grouped, partially grouped, and ungrouped) object configurations were presented, which were identical to the configurations presented in Experiments 1a and 2 and which were presented in a random trial sequence. Response accuracy was stressed, and the responses were not timed. The experiment started with one block of 36 practice trials, followed by nine experimental blocks of 72 trials each, yielding a total of 684 trials.

### Results

**Fig. 7:**
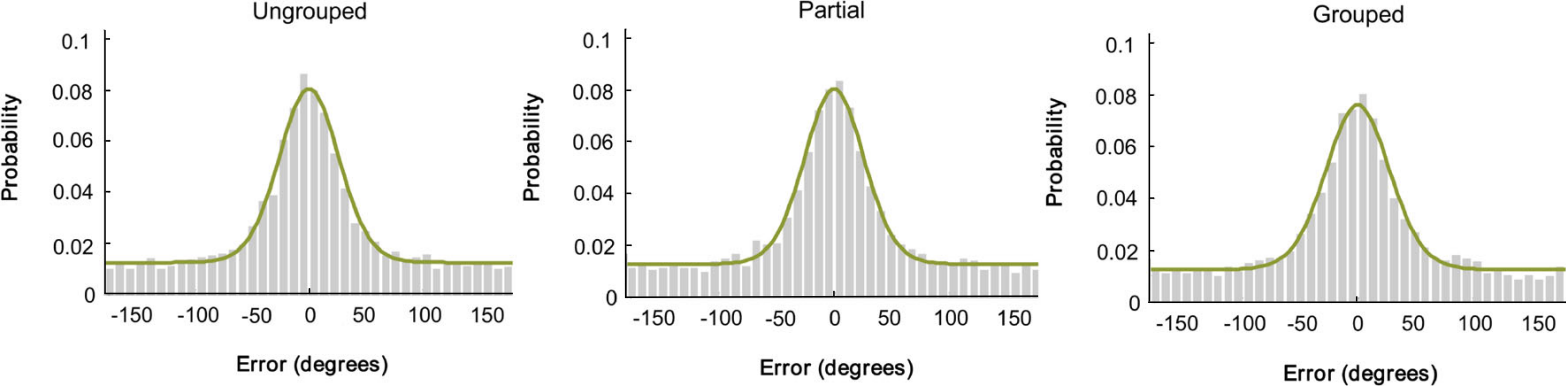
Mean frequency histograms of the response offsets for each object configuration (ungrouped, partially grouped and grouped) in Experiment 3

In this experiment, the data from a given observer consist of a set of distances of the reported from the original color value in each condition (ranging from –180 to +180 degrees). To assess memory performance, we created frequency histograms of response offsets for each object configuration (see Fig. 7). Analyses then focused on the mean absolute offsets (i.e., the distance between the reported and the original color values). Additionally, memory performance was quantified using a standard mixture model (Zhang & Luck, 2008) describing the error distribution in terms of a weighted sum of two distinct distributions: (a) a Gaussian-like distribution (defined on a circular space in terms of a von Mises distribution), assumed to reflect successful memory retrieval with some variable degree of precision; and (b) a uniform distribution reflecting random guessing. Maximum likelihood estimation was used to fit the mixture model to the distribution of response offsets. Two parameters were estimated: the fidelity (precision) of a given memory representation and the probability of guessing. Memory fidelity was estimated as the standard deviation (*SD*) of the von Mises distribution: The narrower the distribution (with a relatively small standard deviation from 0°), the more precise the memory representation. And the probability of guessing (1 − *P*_m_) was estimated by the height of the uniform distribution, with larger values denoting a higher probability of guessing.

A repeated-measures ANOVA on the response offsets, with the factor object configuration (grouped, partially grouped, ungrouped), revealed no significant effect, *F*(2, 38) = 0.28, *p* = .76, η_p_^2^ = .01, 90% CI [0, 0.08], BF_10_ = 0.16 (grouped: 55.49, partially grouped: 55.02, ungrouped: 54.68). Likewise, an analysis of the partially grouped configuration revealed no difference between items located inside and outside of the grouped triangle, *t*(19) = 0.63, *p* = .54, *d*_z_ = .14, BF_10_ = 0.28 (inside: 55.57, outside: 54.47).

**Fig. 8:**
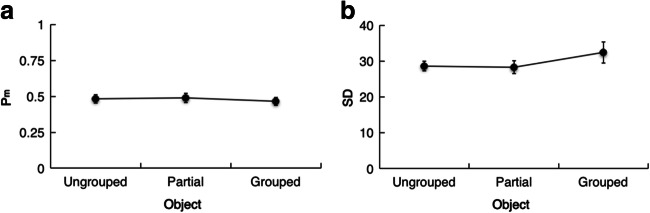
Estimated mean *P*_m_ (**a**) and standard deviation (*SD*) (**b**) parameters from Experiment 3. Error bars show the within-subject standard errors of the means

Figure 8 presents the two estimated parameters, *P*_m_ (Fig. 8a) and *SD* (Fig. 8b), as a function of object configuration. Two repeated-measures ANOVAs on the *P*_m_ and *SD* parameters, with the factor object configuration, again revealed no significant effects: *P*_m_, *F*(2, 38) = .47, *p* = .63, η_p_^2^ = .02, 90% CI [0, .11], BF_10_ = .19; *SD*, *F*(2, 38) = 1.55, *p* = .23, η_p_^2^ = .08, 90% CI [0, .20], BF_10_ = .45. Together, this shows that neither the precision nor the rate of guessing was modulated by variations in the strength of spatial grouping.

### Discussion

Experiment 3 was designed to test whether making the task more demanding with respect to the task-relevant but grouping-irrelevant color features (by requiring participants to memorize item colors with high precision) would induce participants to make effective use of the orientation-based grouping cues. However, despite the change of task and the attendant increase in demands, Experiment 3 replicated the basic finding from the color condition of Experiment 2—that is, absence of an orientation-based grouping benefit when only color information is directly task relevant. The analyses of the response offsets, and the corresponding estimates of parameters *P*_m_ and *SD*, failed to reveal a reliable improvement in color reproduction with an increase in grouping strength. We also did not find any benefit for the inside-triangle locations in the partially grouped memory displays, thus again replicating Experiment 2. This pattern further supports the view that an effect of grouping in VWM depends on the actual task: grouping-irrelevant features are not integrated automatically into a complete-object representation in VWM; rather, a memory benefit is engendered only if the grouping-relevant feature is task-relevant—whatever the degree of detail required by the task at hand or the resolution of the dependent performance measure.

## General discussion

The present study investigated whether the short-term retention of separate features can benefit from spatial grouping that integrates isolated parts into a coherent, whole-object representation. Specifically, the grouping-relevant feature “orientation” and the grouping-irrelevant feature “color” were tested. In Experiments 1a and 1b, both orientation and color changes were intermixed within trial blocks—that is, both were task-relevant and had to be remembered. The results revealed a large grouping benefit for detecting orientation changes; additionally, there was a smaller, but still reliable benefit for color changes. Furthermore, as indicated by Experiment 1b, the observed benefit in memorizing the (grouping-relevant) orientation features is attributable to two separable aspects of perceptual organization: (i) the integration of disparate parts into a coherent object (i.e., object grouping), and (ii) the structuring of individual items into a regular and symmetric layout (i.e., configural regularity)—which both contributed to the improvement in the detection of orientation changes (whereas the detection of color changes benefited reliably from grouping but not reliably from regularity). By contrast, in Experiment 2, in which the orientation and color features were to be encoded and remembered separately, in different trial blocks, there was no longer a grouping benefit for color changes, only one for orientation changes. Experiment 3 further showed that, even when participants had to memorize color information with high resolution and a fine-grained measure was used to assess memory accuracy, grouping (by orientation) still did not facilitate memory for color features. Taken together, these results indicate that both object grouping and configural regularity (i.e., symmetry) can improve VWM performance for orientation changes. However, a benefit of grouping does not necessarily involve the storage of integrated, object-based representations for all—grouping-relevant and grouping-irrelevant—features of a given object. Rather, separate features appear to benefit to variable degrees from grouping—partly depending on the specific attentional set for whole objects versus specific features. Moreover, an object-based enhancement of memory performance appears to be tied to features that give rise to grouping, but these features are represented relatively independently from other features of the same emerging object.

In general, our study replicates previous findings of improved memory performance when multiple features can be represented as a coherent (grouped) object, as compared with when the same set of features is distributed across multiple, separate items (e.g., Fougnie et al., 2013; Olson & Jiang, 2002; Xu, 2002). However, this does not mean that VWM necessarily stores integrated object representations. When both types of feature were task-relevant, separable features revealed an object benefit, but this benefit differed quite substantially between features that did, versus features that did not, determine the grouping. Moreover, when the task demands were changed such that only one feature was to-be-memorized at a time, a VWM benefit was rendered only by the grouping-relevant, but not the grouping-irrelevant feature. We take this to indicate that the representations stored in VWM are feature-specific and separate features may be stored independently of each other (see also Bays et al., 2011; Fougnie & Alvarez, 2011; Fougnie et al., 2013; Pasternak & Greenlee, 2005).

**Fig. 9:**
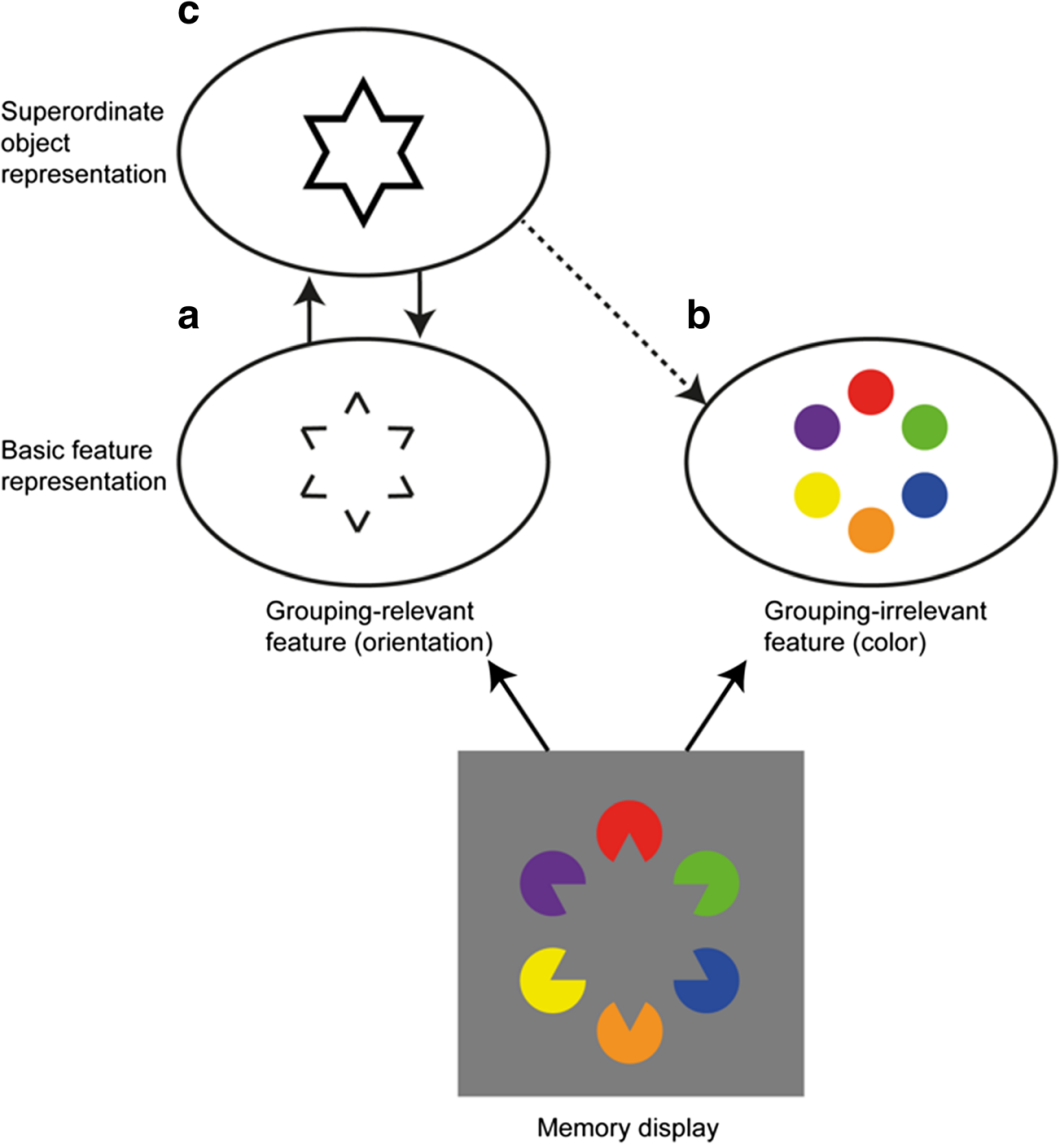
Proposed structure of a hierarchical memory representation with separate grouping-relevant and grouping-irrelevant features and a superordinate, higher-order representation of the grouped object. In the example, a memory display is presented that shows a grouped Kanizsa star configuration as the to-be-memorized input. Both grouping-relevant (orientation) features (**a**) and grouping-irrelevant (color) features (**b**) are represented independently of each other at a basic level, and the grouping-relevant feature additionally gives rise to the formation of a superordinate, grouped object representation (**c**) (note that there is no corresponding superordinate representation which is engendered by the grouping-irrelevant feature). The superordinate object representation in turn enhances the representation of the basic-level grouping-relevant features, and, when task-relevant, it might also (to a certain extent) benefit the representation of the grouping-irrelevant features (dashed line). (Color figure online)

Perceptual grouping provides an efficient means to combine multiple elements into higher-order units. That is, objects might be stored in VWM in terms of a hierarchical structure, comprising basic feature-level representations and associated higher-order, object-level representations (Brady et al., 2011; Nie et al., 2017). Furthermore, we propose that the grouping cue enhances VWM for grouping-relevant features by integrating these (but not the grouping-irrelevant features) into a higher-order, superordinate representation (see also Brady, Konkle, & Alvarez, 2009; Gao et al., 2016); the grouping-irrelevant feature may also benefit from the superordinate object representation via a feedback connection that is enabled only when attention is set to process whole objects. The architecture of such a VWM system is illustrated in Fig. 9: while both the grouping-relevant and grouping-irrelevant features of the to-be-remembered items are represented independently of each other at a basic level (Fig. 9a and b, respectively), encoding of the grouping-relevant features gives rise to the representation of a grouped object at a higher level in the hierarchical structure (see Fig. 9c; see also Chen et al., 2019). That is, the grouped shape is represented as a separate, superordinate representation—with reciprocal (feedforward and feedback) connections between the basic-feature and superordinate-object levels, thereby strengthening the representation of the grouping-relevant features themselves. By contrast, the grouping-irrelevant (color) features do not engender a comparable higher-order object structure. However, when attention is set to whole objects, the superordinate object structure (that emerged from the grouping-relevant features) could additionally come to reinforce the representations of the grouping-irrelevant (color) features via a feedback connection (see dashed line in Fig. 9). Thus, this scheme does not reflect a genuine object-based representation of an integrated whole. Instead, object representations are conceived in terms of their separable features that are stored in VWM independently of each other. Moreover, different features might vary in the degree to which they are supported by emerging higher-level object structures via feedback connections. Within this model framework, the representation of information at the different levels in the hierarchical structure jointly determines the capacity and quality of VWM representations. Importantly, on this account, the various features (represented in VWM independently at the basic feature level) are influenced by each other only indirectly, via feedback from the higher-level object representation.

In more detail, when the task demands that both the grouping-relevant and grouping-irrelevant features are to be memorized (i.e., when a change is to be detected in either type of feature, as in Experiments 1a and 1b), the complete object is brought into the (task-set-dependent) “focus of attention” (see, e.g., Oberauer & Hein, 2012; Souza & Oberauer, 2017), in which case both types of feature benefit from grouping—albeit to variable degrees: grouping-irrelevant features benefit less than the grouping-relevant features. In the model, this is captured by a strong feedback link from the superordinate representation to the grouping-relevant features—in particular (or exclusively) those contributing to the higher-level grouping—whereas the link to the (corresponding) grouping-irrelevant features is relatively weak (illustrated by the solid and, respectively, the dashed feedback arrow in Fig. 9). By contrast, when the task exclusively requires a feature to be reported that does not elicit a higher-order object structure (as in Experiments 2 and 3), an emerging higher-order object representation (driven by grouping-relevant features) would not be brought into the (task-based) focus of attention, so that grouping-irrelevant but task-relevant features would not benefit from the grouping. This implies that the representation of a conjunction of separate features depends on the attentional engagement on whole objects, but nevertheless, the underlying representation in VWM is essentially feature based.

Critically, on this account, activation of the superordinate representation is “gated” by the task-set-dependent focus of attention, which determines whether or not any emergent grouping is actively represented in VWM: Only when the task requires, or the task-set includes, selecting the grouping-relevant features is the corresponding, emergent object (automatically) integrated at the superordinate level in the hierarchical VWM representation (see also Chen et al., 2019; Huang, 2020; Rauschenberger & Yantis, 2001; Wiegand et al., 2015; Woodman & Vogel, 2008). Thus, when the task is to memorize whole objects, observers adopt a more global, object-based processing mode, whereby the overall “ensemble” representation also incorporates, or connects to, the grouping-irrelevant features. By contrast, when only a specific feature is task-relevant, a more local, feature-specific processing set is adopted and VWM does not incorporate the superordinate representation. Such a flexible, task-set-dependent representation may well be a factor in explaining the divergent evidence for object-based versus feature-specific VWM representations reported in the literature (e.g., Luck & Vogel, 1997; Vogel et al., 2001; Zhang & Luck, 2008; Fougnie & Alvarez, 2011; Fougnie et al., 2013).^3^

A potential alternative account to explain the grouping benefits for the grouping-irrelevant (color) features observed in Experiments 1a and 1b could be that grouping (by the grouping-relevant features) may permit a given grouping-relevant feature to be stored as a compressed representation, thus saving memory resources (Brady et al., 2009), which, in turn, could be made available for the representation of the other, grouping-irrelevant feature (see also Morey, 2019). In this view, the integrated object structure facilitates the encoding and/or representation of the grouping-irrelevant feature via the release of memory resources that become available from the compressed (grouped) object, albeit to a lesser degree. This account was directly tested by reexamining the performance for the partially grouped configuration in Experiment 1a. Recall that in this configuration, only three of the Pac-Man inducers gave rise to a (superordinate) Kanizsa triangle—that is, these three (“inside-triangle”) Pac-Man figures could be grouped to form a higher-order structure, while the other three (“outside-triangle”) Pac-Man figures were not integrated into a corresponding grouped shape. Now, memorizing the Pac-Man figures outside of the triangle structure should benefit from the resources released by the compressed, that is, grouped, structure in the same display. However, a comparison of memory performance for trials that presented outside-triangle changes in the partially grouped configuration versus “baseline” trials that presented a completely ungrouped configuration revealed no difference in performance, for either orientation changes (56.2% vs. 59.7%), *t*(19) = −1.40, *p* = .91, *d*_*z*_ = −0.31, BF_10_ = 0.11, or color changes (70% vs. 70.6%), *t*(19) = −0.35, *p* = .64, *d*_*z*_ = −0.08, BF_10_ = 0.18. This indicates that the grouped triangle structure did actually not release memory resources that could then be used to better represent the ungrouped parts (“outside” the triangle) of that very same configuration—thus arguing against an account that assumes flexible resource sharing, whether (only) within the same, grouping-relevant (orientation) features or within different, grouping-irrelevant (color) features. Accordingly, this supplementary analysis provides no evidence for the alternative, “resource-transfer” explanation. Instead, it supports the view that features are represented separately from each other, in feature-specific modules, and grouping benefits are limited to those grouping-relevant features (orientation) that actually determine the integrated object, which benefit robustly, and, to a weaker extent, the corresponding grouping-irrelevant features (color) given the latter are relevant to the task at hand.

Yet another, in some sense related, account would assume that the observed benefit of grouping, rather than reflecting more efficient storage of information in VWM, arises at a perceptual level—that is, from a modulatory effect of perceptual grouping on the allocation of attention. For instance, grouping can influence early processes of attentional selection, with attention in turn facilitating processing of the grouped items (e.g., Marini & Marzi, 2016; Rauschenberger & Yantis, 2001; Senkowski et al., 2005; Wiegand et al., 2015)—in particular, conferring an advantage to grouped items during the encoding of the stimulus configurations into VWM. Attentionally enhanced VWM encoding of grouped items could explain why performance was better for “inside-triangle” versus “outside-triangle” items in our partial-grouping displays (see also Li et al., 2018). However, it fails to explain why fully grouped displays brought about a substantial performance benefit relative to ungrouped configurations—as in both conditions, all items were task-relevant and thus to be attended (i.e., in both conditions, attention should have been distributed equally across all to-be-memorized items). Moreover, on object-based accounts of attentional selection and encoding into VWM, by definition all features characterizing an “object” would be encoded in their entirety (e.g., Peters, Kaiser, Rahm, & Bledowski, 2015; but see, e.g., Müller & O’Grady, 2000), with equal weight, in particular when all are task relevant (see Foerster & Schneider, 2018). However, the asymmetric, larger grouping benefit we found for the detection of orientation as compared with color changes would be at variance with an object-based attentional enhancement of VWM representations.

### Conclusion

The present study demonstrates that VWM representations are essentially feature-specific and benefits deriving from integrated objects actually depend on the specific (task-based) attentional set. If the whole object is task-relevant, grouping of one feature may indirectly also benefit another, ungrouped—yet (in terms of its position within the figural structure) linked—feature. However, when attention is set to only process a specific grouping-irrelevant feature, then no evidence for an overall object-benefit is revealed, suggesting that separate features may be stored independently from each other in VWM.

## Footnotes

1 Change-detection performance usually turns out worse when tested with single-item probe displays as compared with variants that present the entire configuration (possibly including a changed element) again in the probe display (see Jiang, Olson, & Chun, 2000)—the reason being that with whole-configuration probe displays, the additional cues provided by the complete set of items aid the decision on a response. Accordingly, in the current context, the use of single-item probes has the advantage that any effect that might arise from the specific configuration of the display can only be attributable to processes relating to the encoding and the retention of information in VWM, rather to processes related to response decisions.

2 Analogous analyses were also performed on the sensitivity score *d'* and on K, which is typically used to estimate working-memory capacity (Rouder, Morey, Morey, & Cowan, 2011). These additional analyses revealed exactly the same pattern of results as reported for the (% correct) accuracy data, for all experiments.

3 Note that this (critical) assumption does not mean that when the task is solely directed to the grouping-irrelevant feature, giving rise to a purely feature-based representation in VWM, the object itself would not be grouped at a lower, perceptual level. Rather, there is evidence that attending to just some part(s) of an object triggers perceptual completion operations irrespective of the specific task set (Chen, Weidner, Heng, Fink, Müller, & Conci, 2020; see also Chen et al., 2019; Conci, Groß, Keller, Müller, & Finke, 2018; Gögler, Finke, Keller, Müller, & Conci, 2016).

## References

[CR1] Bays PM, Wu EY, Husain M (2011). Storage and binding of object features in visual working memory. Neuropsychologia.

[CR2] Brady TF, Konkle T, Alvarez GA (2009). Compression in visual working memory: Using statistical regularities to form more efficient memory representations. Journal of Experimental Psychology: General.

[CR3] Brady, T. F., Konkle, T., & Alvarez, G. A. (2011). A review of visual memory capacity: Beyond individual items and toward structured representations. *Journal of Vision, 11*(5), 4, 1–34. 10.1167/11.5.4 10.1167/11.5.4 PMC3405498 21617025

[CR4] Brainard DH (1997). The Psychophysics Toolbox. Spatial Vision.

[CR5] Chen S, Glasauer S, Müller HJ, Conci M (2018). Surface filling-in and contour interpolation contribute independently to Kanizsa figure formation. Journal of Experimental Psychology: Human Perception and Performance.

[CR6] Chen S, Müller HJ, Conci M (2016). Amodal completion in visual working memory. Journal of Experimental Psychology: Human Perception and Performance.

[CR7] Chen S, Nie Q-Y, Müller HJ, Conci M (2019). Kanizsa-figure object completion gates selection in the attentional blink. Quarterly Journal of Experimental Psychology.

[CR8] Chen S, Töllner T, Müller HJ, Conci M (2018). Object maintenance beyond their visible parts in working memory. Journal of Neurophysiology.

[CR9] Chen S, Weidner R, Zeng H, Fink GR, Müller HJ, Conci M (2020). Tracking the completion of parts into whole objects: Retinotopic activation in response to illusory figures in the lateral occipital complex. NeuroImage.

[CR10] Conci M, Groß J, Keller I, Müller HJ, Finke K (2018). Attention as the “glue” for object integration in parietal extinction. Cortex.

[CR11] Conci M, Müller HJ, Elliott MA (2007). The contrasting impact of global and local object attributes on Kanizsa figure detection. Perception & Psychophysics.

[CR12] Delvenne J, Bruyer R (2004). Does visual short-term memory store bound features?. Visual Cognition.

[CR13] Delvenne J-F, Bruyer R (2006). A configural effect in visual short-term memory for features from different parts of an object. Quarterly Journal of Experimental Psychology.

[CR14] Ecker UKH, Maybery M, Zimmer HD (2013). Binding of intrinsic and extrinsic features in working memory. Journal of Experimental Psychology: General.

[CR15] Erdfelder E, Faul F, Buchner A (1996). G*Power: A general power analysis program. Behavior Research Methods, Instruments, & Computers: A Journal of the Psychonomic Society, Inc.

[CR16] Foerster RM, Schneider WX (2018). Involuntary top-down control by search-irrelevant features: Visual working memory biases attention in an object-based manner. Cognition.

[CR17] Fougnie, D., & Alvarez, G. A. (2011). Object features fail independently in visual working memory: Evidence for a probabilistic feature-store model. *Journal of Vision, 11*(12). 10.1167/11.12.3 10.1167/11.12.3 PMC3279121 21980189

[CR18] Fougnie D, Cormiea SM, Alvarez GA (2013). Object-based benefits without object-based representations. Journal of Experimental Psychology: General.

[CR19] Gao T, Gao Z, Li J, Sun Z, Shen M (2011). The perceptual root of object-based storage: An interactive model of perception and visual working memory. Journal of Experimental Psychology: Human Perception and Performance.

[CR20] Gao Z, Gao Q, Tang N, Shui R, Shen M (2016). Organization principles in visual working memory: Evidence from sequential stimulus display. Cognition.

[CR21] Gögler N, Finke K, Keller I, Müller HJ, Conci M (2016). Object integration requires attention: Visual search for Kanizsa figures in parietal extinction. Neuropsychologia.

[CR22] Huang L (2020). Unit of visual working memory: A Boolean map provides a better account than an object does. Journal of Experimental Psychology: General.

[CR23] Jeffreys H (1961). Theory of probability.

[CR24] Jiang Y, Olson IR, Chun MM (2000). Organization of visual short-term memory. Journal of Experimental Psychology: Learning, Memory, and Cognition.

[CR25] Kanizsa G (1955). Margini quasi-percettivi in campi con stimolazione omogenea. Rivista di psicologia.

[CR26] Kass RE, Raftery AE (1995). Bayes factors. Journal of the American Statistical Association.

[CR27] Li J, Qian J, Liang F (2018). Evidence for the beneficial effect of perceptual grouping on visual working memory: An empirical study on illusory contour and a meta-analytic study. Scientific Reports.

[CR28] Love, J., Selker, R., Marsman, M., Jamil, T., Dropmann, D., Verhagen, A. J., & Wagenmakers, E. J. (2015). JASP (Version 0.7)[computer software]. Amsterdam, the netherlands: Jasp project

[CR29] Luck SJ, Vogel EK (1997). The capacity of visual working memory for features and conjunctions. Nature.

[CR30] Luria R, Vogel EK (2011). Shape and color conjunction stimuli are represented as bound objects in visual working memory. Neuropsychologia.

[CR31] Ma WJ, Husain M, Bays PM (2014). Changing concepts of working memory. Nature Neuroscience.

[CR32] Magnussen S, Greenlee MW (1999). The psychophysics of perceptual memory. Psychological Research.

[CR33] Marini F, Marzi CA (2016). Gestalt perceptual organization of visual stimuli captures attention automatically: Electrophysiological evidence. Frontiers in Human Neuroscience.

[CR34] Morey CC (2019). Perceptual grouping boosts visual working memory capacity and reduces effort during retention. British Journal of Psychology.

[CR35] Morey CC, Cong Y, Zheng Y, Price M, Morey RD (2015). The color-sharing bonus: Roles of perceptual organization and attentive processes in visual working memory. Archives of Scientific Psychology.

[CR36] Müller HJ, O’Grady RB (2000). Dimension-based visual attention modulates dual-judgment accuracy in Duncan’s (1984) one- versus two-object report paradigm. Journal of Experimental Psychology: Human Perception and Performance.

[CR37] Nie Q-Y, Müller HJ, Conci M (2017). Hierarchical organization in visual working memory: From global ensemble to individual object structure. Cognition.

[CR38] Oberauer K, Hein L (2012). Attention to information in working memory. Current Directions in Psychological Science.

[CR39] Olson IR, Jiang Y (2002). Is visual short-term memory object based? Rejection of the “strong-object” hypothesis. Perception & Psychophysics.

[CR40] Pasternak T, Greenlee MW (2005). Working memory in primate sensory systems. Nature Reviews. Neuroscience.

[CR41] Peters B, Kaiser J, Rahm B, Bledowski C (2015). Activity in human visual and parietal cortex reveals object-based attention in working memory. The Journal of Neuroscience.

[CR42] Peterson DJ, Berryhill ME (2013). The Gestalt principle of similarity benefits visual working memory. Psychonomic Bulletin & Review.

[CR43] Quinlan PT, Cohen DJ (2012). Grouping and binding in visual short-term memory. Journal of Experimental Psychology: Learning, Memory, and Cognition.

[CR44] Rauschenberger R, Yantis S (2001). Attentional capture by globally defined objects. Perception & Psychophysics.

[CR45] Rouder JN, Morey RD, Morey CC, Cowan N (2011). How to measure working memory capacity in the change detection paradigm. Psychonomic Bulletin & Review.

[CR46] Senkowski D, Röttger S, Grimm S, Foxe JJ, Herrmann CS (2005). Kanizsa subjective figures capture visual spatial attention: Evidence from electrophysiological and behavioral data. Neuropsychologia.

[CR47] Souza AS, Oberauer K (2017). The contributions of visual and central attention to visual working memory. Attention, Perception, & Psychophysics.

[CR48] Vogel EK, Woodman GF, Luck SJ (2001). Storage of features, conjunctions and objects in visual working memory. Journal of Experimental Psychology: Human Perception and Performance.

[CR49] Wang B, Cao X, Theeuwes J, Olivers CNL, Wang Z (2017). Separate capacities for storing different features in visual working memory. Journal of Experimental Psychology: Learning Memory and Cognition.

[CR50] Wheeler ME, Treisman AM (2002). Binding in short-term visual memory. Journal of Experimental Psychology: General.

[CR51] Wiegand I, Finke K, Töllner T, Starman K, Müller HJ, Conci M (2015). Age-related decline in global form suppression. Biological Psychology.

[CR52] Woodman GF, Vecera SP, Luck SJ (2003). Perceptual organization influences visual working memory. Psychonomic Bulletin & Review.

[CR53] Woodman GF, Vogel EK (2008). Selective storage and maintenance of an object’s features in visual working memory. Psychonomic Bulletin & Review.

[CR54] Xu Y (2002). Encoding color and shape from different parts of an object in visual short-term memory. Perception & Psychophysics.

[CR55] Zhang W, Luck SJ (2008). Discrete fixed-resolution representations in visual working memory. Nature.

